# Supplementary material for: Kynurenine Pathway of Tryptophan Metabolism Is Associated with Hospital Mortality in Patients with Acute Respiratory Distress Syndrome: A Prospective Cohort Study
Source: Antioxidants (Basel). 2022 Sep 23;11(10):1884. doi: 10.3390/antiox11101884 (PMC9598717; doi:10.3390/antiox11101884)
Supplement: Supplementary file 1 [file antioxidants-11-01884-s001.zip › antioxidants-1876803-supplementary.pdf]

Supplementary Information

Kynurenine Pathway of Tryptophan Metabolism is Associated with Hospital Mortality in Patients with Acute Respiratory Distress Syndrome: A Prospective Cohort Study

Li-Chung Chiu, Hsiang-Yu Tang, Chun-Ming Fan, Chi-Jen Lo, Han-Chung Hu, Kuo-Chin Kao, Mei-Ling Cheng

1. **Supplementary Methods**

2. **Supplementary Tables**

2.1 Table S1 Concentrations of plasma amino acids: healthy controls and ARDS patients.

2.2 Table S2 Concentrations of plasma amino acids: survivors and nonsurvivors of ARDS patients.

2.3 Table S3 Concentrations of plasma biogenic amines: healthy controls and ARDS patients.

2.4 Table S4 Concentrations of plasma biogenic amines: survivors and nonsurvivors of ARDS patients.

1. Supplementary Methods

Sample Collection and Preparation

Plasma and urine samples were obtained from patients with acute respiratory distress syndrome (ARDS) after fasting overnight (at least 8 h) at day 1, day 3, and day 7 after ARDS onset. Plasma and urine samples were also obtained from healthy controls living in the Chang Gung Health and Culture Village in Taoyuan, Taiwan. The controls had no significant comorbidities and were asked to fast overnight (at least 8 h).

Whole blood (10 ml) was collected under sterile conditions in EDTA-coated tubes and subjected to centrifugation within 2 h (3,000 *rpm*; 4°C; 10 min), whereupon the plasma was transferred into a 15 mL polypropylene tube. Cell debris was removed from the plasma via centrifugation at 3,000 *rpm* at 4°C for 10 min. After the second centrifugation, the plasma was aliquoted in an Eppendorf tube and stored at –80°C until the time of assay. Urine samples were centrifuged at 3,000 *rpm* at 4°C for 10 min, whereupon the supernatant was collected in an Eppendorf tube and stored at –80°C until the time of assay.

Bronchoalveolar lavage was collected via a bronchoscope examination at day 1; however, it was not performed in cases where the fraction of inspired oxygen was ≧ 0.8. Sterile normal saline (50 ml; room temperature) was injected via a handheld syringe

through a flexible bronchoscope. This process was repeated three to five times, such that a total of 250 ml was instilled. The instilled fluid was then aspirated under gentle suctioning through the suction port of the bronchoscope back into the syringe. The area in which the bronchoscope was wedged depended on whether the disease was localized or diffuse. In cases where disease localization predominated (as identified using high-resolution computed tomography or chest X-ray), the bronchoscope was guided to the specific region and wedged into that subsegment of the lungs. In cases of bilaterally diffuse infiltrating disease, the preferred location for lavage was the right middle lobe or lingula. Bronchoalveolar lavage fluid was centrifuged at 2,000 *rpm* at 4°C for 5 min, whereupon the supernatant has been stored at –80°C until the time of assay (not yet analyzed in this study).

Metabolomics Analysis of Amino Acids and Biogenic Amines in Plasma and Urine Samples

We followed general precautions pertaining to sample collection and storage (before and after extraction) to ensure that only high-quality information was obtained [43, 44]. Metabolomic analysis was conducted in accordance with standardized procedures in the Metabolomic Core Laboratory at Chang Gang University in Taoyuan, Taiwan.

Chemicals and Reagents

Amino acid standards, including histidine, asparagine, taurine, serine, glutamine, arginine, glycine, aspartic acid, citrulline, glutamic acid, threonine, alanine, proline, ornithine, cysteine, lysine, tyrosine, methionine, valine, isoleucine, leucine, phenylalanine, tryptophan, and norvaline, were purchased from Sigma-Aldrich (St. Louis, MO, USA). 5-sulfosalicylic acid dehydrate, boric acid, sodium tetraborate, ammonium formate, and sodium hydroxide were purchased from Sigma-Aldrich (St. Louis, MO, USA). 6-aminoquinolyl-N-hydroxysuccinimidyl carbamate (AQC) was purchased from Gold Biotechnology (Inc., St. Louis, MO). Biogenic amine commercial standard, 96-well sample collection plates, 96-well sample filter plates, and specific columns for amino acid analysis were purchased from Waters (Milford, USA).

Amino Acid Analysis of Plasma Samples Using Ultra-high Performance Liquid Chromatography (UPLC)

Plasma samples were collected and stored at –80°C until the time of assay. Plasma samples (100 μl) underwent protein precipitation by adding an equal volume of 10% sulfosalicylic acid containing an internal standard (norvaline, 200 μM). The samples were then vortexed and centrifuged at 12,000*g* at 4°C for 10 min. After centrifugation, 20 μl of the supernatant was mixed with 60 μl of borate buffer (pH 8.8). Derivatization was initiated by adding 20 μl of 10 mM AQC in acetonitrile. After incubation for 10 min, the mixture was combined with 100 μl of 20 mM ammonium formate/0.8% formic acid/1% acetonitrile) and transferred into

vials to be placed in a refrigerated autosampler in a UPLC system at 10°C. Note that the Waters ACQUITY UPLC System comprised a Binary Solvent Manager (BSM), an autosampler, and a photodiode array (PDA) detector. Separations were performed using an ACQUITY BEH C18 column (2.1 × 100-mm i.d., 1.7 μm, Waters Corp., Milford, USA) at 55°C using mobile phase A (20 mM ammonium formate/0.8% formic acid/1% acetonitrile) and mobile phase B (acetonitrile) at a flow rate of 0.6 ml/min with a linear gradient. The initial composition was maintained at 0.1%B between 0–2.5 min, 2%B between 2.5–3.2 min, 9%B between 3.2–4.1 min, 10%B between 4.1–4.9 min, held for 1 min, and then 20%B between 5.9–7min. The column was then washed with 90%B for 1 min and re-equilibrated for 0.7 min. Amino acids were detected using a PDA detector at a wavelength of 280 nm.

Biogenic Amine Analysis of Plasma Samples Using Liquid Chromatography-mass Spectrometry (LCMS)

Plasma samples were collected and stored at -80°C until the time of assay. 100 μl of 10% sulfosalicylic acid containing an internal standard and an equal volume (100 μl) of plasma were added to a 96-well plate with a filter membrane for protein precipitation. Samples were centrifuged at 2,000*g* at 4 °C for 10 min. Subsequently, 100 μl of the filtrate was acidified using 900 μl of 0.1 N hydrochloric acid, whereupon 20 μl of the mixture was mixed with 60 μl of borate buffer and 20 μl of AQC for

derivatization. After incubation for 10 min, the mixture was combined with 900 µl of water containing 0.1% formic acid for LC-MS/MS analysis [45]. Chromatographic separation was achieved using a CORTECS C18 reversed-phase column (2.1 × 150-mm i.d., 1.6 µm, Waters Corp., Milford, USA) at 55°C with mobile phase A (0.1% formic acid in water, v/v) and mobile phase B (0.1% formic acid in acetonitrile, v/v) with the flow rate set at 0.5 ml/min. The gradient profile was as follows: equilibrate with 1%B for 1 min, then linear gradients of 1–13%B for 1 min, 13–15%B for 3.5 min, 15–95%B for 1 min, and hold at 95%B for 1 min, then back to 1%B for 0.1 min and maintain at 1%B for 1.4 min to recondition the column. The MSMS parameters were as follows: capillary voltage 2 kV, desolvation gas flow 1,000 L/h, desolvation temperature 500°C, source temperature 150°C, and voltage 20 V. System operation and data acquisition were controlled using Mass Lynx software, and targeted metabolic data were analyzed using TargetLynx (Waters, Milford, USA).

Urine Sample Preparation for Global Analysis of Metabolites

Urine samples were diluted using distilled water to a creatinine content of 100 µg/ml. Samples are centrifuged at 14,000*g* for 30 minutes, whereupon the supernatant was collected for LC-MS analysis.

Global Metabolite Profiling Analysis of Urine Samples

Liquid chromatographic separation was achieved using a 100 mm × 2.1 mm, 1.8 μm, Acquity HSS T3 column (Waters Corp; Milford, MA, USA) with an ACQUITY TM Ultra Performance Liquid Chromatography system (Waters Corp; Milford, MA, USA) [46]. The column was maintained at 40°C with a flow rate of 0.5 ml/min. Samples were eluted from the LC column with a linear gradient, as follows: 1–15%B between 0–2 min, 15–50%B between 2–5 min, 50–98%B between 5–6.5 min, 98%B between 6.5–8 min, and 1%B between 8.1–10 min for re-equilibration. Mobile phase A was water containing 0.1% formate and mobile phase B was acetonitrile containing 0.1% formate. Each sample was analyzed three times.

Mass Spectrometry

Mass spectrometry was performed using a Waters QTOF MS (SYNAPT HDMS, Waters MS Technologies, Manchester, UK) operated in positive ion mode with a scanning range of 20 to 1,000 m/z. The desolvation gas was set to 1,000 l/h at a temperature of 400°C, while the cone gas was set to 25 l/h with the source temperature set at 120°C. The capillary voltage was

set to 2,500 V for ESI positive mode and 2,000 V for ESI negative mode, and the cone voltage was set to 35 V. Leucine enkephalin was used as the lock mass (an $[M+H]^+$ ion at 556.2771 Da in ESI positive mode with an $[M+H]^-$ ion at 554.2615 Da in ESI negative mode).

Metabolomic Software – Markerlyn XS

All information related to MS data, including retention times, m/z, and ion intensities, were extracted using Progenesis QI v2.4 software (Nonlinear Dynamics, Waters, Newcastle, UK), whereupon the resulting MS data were assembled into a matrix. The search for metabolites was performed using METLIN (https://metlin.scripps.edu) and human metabolome (https://hmdb.ca) databases or/and confirmed using standard samples based on retention times as well as mass spectra. The data were then analyzed using principal component analysis (PCA) and orthogonal partial least squares discriminant analysis (OPLS-DA) using SIMCA 13.0 (Umetrics, Umeå, Sweden) (Satorius, Göttingen, Germany) and the MetaboAnalyst 5.0 platform (https://www.metaboanalyst.ca) [47].

Metabolite Identification

The structural identification of target metabolites was performed using standards operated under chromatographic conditions identical to those used in the profiling experiment. MS and MS/MS analyses were performed under the same conditions. MS/MS spectra were collected at 0.1 spectra per second, with a medium isolation window of ~4 m/z. The collision energy was set from 5 to 35 V.

Table S1. Concentrations of plasma amino acids: healthy controls and ARDS patients.

**Metabolites (μM)**	**Healthy Controls (*n* = 30)**	**ARDS Day 1 (*n* = 69)**	**ARDS Day 3 (*n* = 69)**	**ARDS Day 7 (*n* = 69)**	p^1	p^2	p^3
Histidine	80.4 ± 12.4	66.7 ± 20.5	65.2 ± 25.2	70.3 ± 45.5	0.001	0.002	0.238
Asparagine	54.7 ± 9.7	43.9 ± 18.3	50.7 ± 23.0	61.9 ± 58.4	<0.001	0.227	0.358
Taurine	63.7 ± 20.6	37.8 ± 22.8	34.4 ± 17.0	53.2 ± 58.6	<0.001	<0.001	0.345
Serine	108.7 ± 14.3	62.7 ± 26.8	71.8 ± 28.2	83.8 ± 45.3	<0.001	<0.001	<0.001
Glutamine	558.5 ± 80.3	409.5 ± 138.1	455.5 ± 136.7	512.7 ± 327.3	<0.001	<0.001	0.453
Arginine	74.8 ± 16.8	51.7 ± 30.2	61.0 ± 32.8	71.2 ± 74.0	<0.001	0.007	0.795
Glycine	229.4 ± 62.1	154.8 ± 55.8	176.8 ± 86.5	220.7 ± 167.7	<0.001	0.003	0.783
Aspartate	1.8 ± 1.1	3.5 ± 3.2	3.2 ± 2.8	5.2 ± 6.6	0.026	0.141	0.029
Citrulline	38.3 ± 7.4	23.2 ± 17.3	23.2 ± 16.9	33.8 ± 41.2	<0.001	<0.001	0.439
Glutamate	43.5 ± 17.5	91.3 ± 70.9	107.2 ± 62.3	138.8 ± 97.9	<0.001	<0.001	<0.001
Threonine	121.7 ± 22.8	95.8 ± 50.5	129.6 ± 54.4	156.9 ± 109.2	0.001	0.313	0.018
Alanine	424.4 ± 99.4	278.8 ± 121.2	309.0 ± 216.9	359.0 ± 334.4	<0.001	0.007	0.299
Proline	216.5 ± 88.9	200.0 ± 111.8	243.6 ± 130.9	289.8 ± 266.5	0.476	0.303	0.147
Ornithine	88.0 ± 18.0	86.2 ± 47.2	96.3 ± 36.0	123.4 ± 83.8	0.783	0.133	0.002
Lysine	230.7 ± 33.6	183.7 ± 74.8	229.1 ± 99.7	286.9 ± 279.7	<0.001	0.908	0.126
Tyrosine	55.6 ± 11.1	77.8 ± 29.7	85.9 ± 38.2	110.1 ± 132.7	<0.001	<0.001	0.002
Methionine	27.8 ± 3.7	25.0 ± 11.3	32.2 ± 23.9	46.5 ± 101.3	0.065	0.142	0.318
Valine	261.2 ± 38.0	220.5 ± 70.2	237.1 ± 82.5	267.8 ± 117.6	<0.001	0.051	0.692

Isoleucine	66.6 ± 14.9	57.7 ± 24.3	68.2 ± 27.3	76.4 ± 46.5	0.028	0.717	0.137
Leucine	125.5 ± 24.2	106.7 ± 37.5	118.5 ± 49.9	135.7 ± 89.2	0.004	0.353	0.412
Phenylalanine	61.8 ± 16.4	108.0 ± 40.8	109.6 ± 57.9	143.0 ± 135.6	<0.001	<0.001	<0.001
Tryptophan	42.3 ± 10.8	25.5 ± 10.5	29.5 ± 11.3	35.9 ± 15.7	<0.001	<0.001	0.045

Values are expressed as mean ± standard deviation. ARDS: acute respiratory distress syndrome.

[1]: Healthy Controls vs ARDS Day 1.

[2]: Healthy Controls vs ARDS Day 3.

[3]: Healthy Controls vs ARDS Day 7.

Table S2. Concentrations of plasma amino acids: survivors and nonsurvivors of ARDS patients.

Metabolites (μM)	ARDS Day 1			ARDS Day 3			ARDS Day 7		
	Survivors (*n* = 33)	Nonsurvivors (*n* = 36)	*p*	Survivors (*n* = 33)	Nonsurvivors (*n* = 36)	*p*	Survivors (*n* = 33)	Nonsurvivors (*n* = 36)	*p*
Histidine	66.5 ± 15.0	66.9 ± 24.7	0.942	63.0 ± 13.2	67.4 ± 32.8	0.474	61.2 ± 10.8	79.7 ± 63.1	0.124
Asparagine	45.6 ± 17.3	42.3 ± 19.3	0.466	52.3 ± 14.1	49.2 ± 29.1	0.581	51.2 ± 13.7	73.4 ± 82.0	0.160
Taurine	37.6 ± 18.5	38.0 ± 26.3	0.933	35.4 ± 17.6	33.5 ± 16.6	0.648	38.0 ± 17.0	69.0 ± 79.3	0.044
Serine	68.3 ± 27.0	57.6 ± 25.8	0.097	79.1 ± 28.0	64.9 ± 27.0	0.037	79.6 ± 21.9	88.2 ± 60.9	0.470
Glutamine	436.6 ± 121.8	384.8 ± 148.8	0.120	469.7 ± 104.4	442.1 ± 161.8	0.409	459.0 ± 105.8	568.1 ± 451.5	0.206
Arginine	56.1 ± 27.8	47.6 ± 32.2	0.251	66.1 ± 33.2	56.1 ± 32.1	0.213	61.6 ± 23.0	81.2 ± 120.9	0.307
Glycine	159.0 ± 49.6	150.9 ± 61.4	0.552	169.8 ± 44.6	183.5 ± 113.1	0.508	194.8 ± 62.9	247.5 ± 229.4	0.233
Aspartate	5.2 ± 3.8	2.2 ± 1.9	0.024	2.9 ± 2.4	3.6 ± 3.3	0.580	2.8 ± 1.9	8.0 ± 8.9	0.097
Citrulline	23.2 ± 18.2	23.2 ± 16.7	0.990	21.5 ± 17.3	25.3 ± 16.5	0.412	26.9 ± 20.2	41.2 ± 55.2	0.206
Glutamate	84.8 ± 55.8	97.2 ± 82.8	0.472	95.5 ± 44.7	118.2 ± 74.2	0.134	123.5 ± 50.2	154.6 ± 129.4	0.226
Threonine	106.1 ± 54.3	86.4 ± 45.6	0.105	136.2 ± 51.6	123.4 ± 56.9	0.333	148.8 ± 44.0	165.3 ± 150.0	0.565
Alanine	267.4 ± 108.7	289.3 ± 132.2	0.456	285.2 ± 86.9	331.4 ± 290.7	0.383	298.4 ± 102.7	421.7 ± 461.0	0.162
Proline	204.6 ± 89.2	195.7 ± 130.3	0.743	246.0 ± 93.2	241.4 ± 160.0	0.885	252.3 ± 86.4	328.6 ± 369.0	0.278
Ornithine	88.4 ± 55.8	84.2 ± 38.5	0.714	96.8 ± 35.8	95.8 ± 36.7	0.912	113.7 ± 47.9	133.5 ± 109.3	0.362
Lysine	195.1 ± 87.1	173.3 ± 60.7	0.229	241.1 ± 95.3	217.8 ± 103.9	0.340	258.6 ± 94.7	316.2 ± 388.3	0.435
Tyrosine	75.5 ± 23.4	79.9 ± 34.7	0.537	81.9 ± 29.3	89.7 ± 45.0	0.402	84.2 ± 36.2	136.8 ± 183.4	0.134
Methionine	27.0 ± 11.4	23.2 ± 11.0	0.164	31.2 ± 11.1	33.2 ± 31.7	0.717	29.2 ± 11.5	64.3 ± 143.0	0.190
Valine	233.9 ± 75.4	208.2 ± 63.7	0.131	247.6 ± 83.4	227.3 ± 81.6	0.316	260.8 ± 83.2	275.0 ± 146.0	0.642

Isoleucine	62.6 ± 27.3	53.2 ± 20.6	0.109	72.2 ± 27.5	64.4 ± 27.1	0.243	75.9 ± 22.9	77.0 ± 62.6	0.924
Leucine	115.6 ± 42.1	98.7 ± 31.2	0.065	125.1 ± 42.1	112.4 ± 56.1	0.299	129.7 ± 42.3	141.8 ± 120.6	0.602
Phenylalanine	106.9 ± 42.2	109.1 ± 40.1	0.830	97.9 ± 35.8	120.6 ± 71.6	0.101	99.7 ± 36.0	187.8 ± 180.6	0.013
Tryptophan	25.7 ± 9.3	25.4 ± 11.6	0.896	30.3 ± 9.9	28.8 ± 12.6	0.572	34.0 ± 11.2	37.7 ± 19.3	0.368

Values are expressed as mean ± standard deviation. ARDS: acute respiratory distress syndrome.

Table S3. Concentrations of plasma biogenic amines: healthy controls and ARDS patients.

Metabolites (μM)	Healthy Controls (*n* = 30)	ARDS Day 1 (*n* = 69)	ARDS Day 3 (*n* = 69)	ARDS Day 7 (*n* = 69)	p^1	p^2	p^3
Ethanolamine	8.4 ± 1.1	8.7 ± 10.5	10.6 ± 14.5	14.6 ± 18.7	0.853	0.209	0.012
Hydroxylysine	1.0 ± 0.5	1.1 ± 0.3	1.2 ± 0.3	1.4 ± 0.6	0.093	0.003	0.001
4-hydroxyproline	20.3 ± 10.4	10.4 ± 5.0	11.6 ± 6.8	14.7 ± 11.4	<0.001	<0.001	0.025
1-Methylhistidine	5.6 ± 1.5	19.6 ± 24.4	18.1 ± 17.2	19.2 ± 21.2	<0.001	<0.001	<0.001
3-Methylhistidine	5.2 ± 5.1	5.3 ± 7.5	3.6 ± 3.3	3.4 ± 2.8	0.953	0.121	0.081
Phosphoethanolamine	6.2 ± 2.4	2.3 ± 1.4	2.2 ± 1.3	2.9 ± 2.4	<0.001	<0.001	<0.001
Anserine	ND	30.0 ± 39.2	29.5 ± 26.8	35.3 ± 40.9	-	-	-
Sarcosine	6.9 ± 1.3	14.4 ± 6.6	14.2 ± 6.2	15.5 ± 9.8	<0.001	<0.001	<0.001
β-Alanine	25.0 ± 2.3	30.8 ± 5.8	31.8 ± 6.6	34.4 ± 10.6	<0.001	<0.001	<0.001
Cystathionine	0.9 ± 0.3	2.8 ± 3.5	3.2 ± 4.2	5.5 ± 13.0	<0.001	<0.001	0.008
α-Aminoadipic acid	2.0 ± 0.4	2.1 ± 1.0	2.4 ± 1.1	3.5 ± 4.1	0.612	0.004	0.005
S-Sulfocysteine	2.0 ± 0.6	4.0 ± 2.3	4.4 ± 2.1	5.2 ± 3.4	<0.001	<0.001	<0.001
γ-Aminobutyric Acid	0.7 ± 0.1	0.5 ± 0.2	0.6 ± 0.4	0.7 ± 0.9	<0.001	0.013	0.639
β-Aminoisobutyric Acid	2.6 ± 1.6	9.7 ± 13.5	9.1 ± 11.7	10.8 ± 14.9	<0.001	<0.001	<0.001
α-Aminobutyric acid	31.6 ± 7.2	19.6 ± 10.8	20.1 ± 11.2	22.1 ± 13.4	<0.001	<0.001	<0.001
Homocitrulline	ND	2.6 ± 3.4	2.2 ± 2.3	2.4 ± 2.6	-	-	-
Homocystine	0.9 ± 0.1	0.7 ± 0.4	0.6 ± 0.3	0.9 ± 1.2	<0.001	<0.001	0.982
Kynurenine	3.7 ± 0.6	25.1 ± 19.1	29.8 ± 25.2	29.3 ± 39.6	<0.001	<0.001	<0.001
Alloisoleucine	3.4 ± 0.9	1.6 ± 1.0	1.6 ± 1.2	1.5 ± 0.9	<0.001	<0.001	<0.001

Values are presented as mean ± standard deviation. ARDS: acute respiratory distress syndrome; ND: not detected.

[1]: Healthy Controls vs ARDS Day 1.

[2]: Healthy Controls vs ARDS Day 3.

[3]: Healthy Controls vs ARDS Day 7.

Table S4. Concentrations of plasma biogenic amines: survivors and nonsurvivors of ARDS patients.

Metabolites (μM)	ARDS Day 1			ARDS Day 3			ARDS Day 7		
	Survivors (n = 33)	Nonsurvivors (n = 36)	p	Survivors (n = 33)	Nonsurvivors (n = 36)	p	Survivors (n = 33)	Nonsurvivors (n = 36)	p
Ethanolamine	7.6 ± 4.2	9.7 ± 13.9	0.413	7.1 ± 2.4	11.7 ± 14.5	0.077	8.9 ± 9.0	20.5 ± 23.9	0.017
Hydroxylysine	1.1 ± 0.3	1.2 ± 0.4	0.328	1.1 ± 0.2	1.3 ± 0.4	0.028	1.2 ± 0.3	1.6 ± 0.7	0.027
4-hydroxyproline	9.1 ± 3.6	11.6 ± 5.7	0.039	9.7 ± 3.4	13.5 ± 8.6	0.021	11.0 ± 5.0	18.5 ± 14.6	0.011
1-Methylhistidine	15.2 ± 15.6	23.7 ± 29.9	0.145	14.5 ± 12.9	21.4 ± 20.1	0.094	12.3 ± 12.2	26.4 ± 25.9	0.010
3-Methylhistidine	4.6 ± 5.8	5.9 ± 8.9	0.497	3.6 ± 3.9	3.6 ± 2.7	0.968	2.6 ± 1.9	4.2 ± 3.4	0.039
Phosphoethanolamine	2.3 ± 1.1	2.3 ± 1.6	0.772	2.2 ± 1.2	2.2 ± 1.4	0.800	2.3 ± 1.4	3.5 ± 3.0	0.059
Anserine	23.2 ± 16.8	36.4 ± 51.4	0.164	31.1 ± 32.7	28.0 ± 20.1	0.639	24.6 ± 21.1	46.4 ± 52.5	0.041
Sarcosine	13.1 ± 4.4	15.6 ± 8.0	0.106	12.7 ± 4.5	15.5 ± 7.2	0.059	13.0 ± 5.4	18.2 ± 12.4	0.042
β-Alanine	31.5 ± 5.8	30.2 ± 5.9	0.350	30.8 ± 7.4	32.8 ± 5.7	0.200	32.7 ± 2.9	36.1 ± 14.7	0.220
Cystathionine	2.3 ± 2.5	3.3 ± 4.3	0.228	2.9 ± 4.7	3.4 ± 3.8	0.632	4.5 ± 15.8	6.4 ± 9.4	0.575
α-Aminoadipic acid	2.0 ± 0.8	2.1 ± 1.2	0.805	2.2 ± 0.9	2.7 ± 1.3	0.038	2.6 ± 1.4	4.5 ± 5.6	0.071
S-Sulfocysteine	3.7 ± 2.1	4.4 ± 2.4	0.187	4.2 ± 2.0	4.5 ± 2.2	0.564	5.5 ± 3.8	4.8 ± 2.9	0.387
γ-Aminobutyric Acid	0.5 ± 0.1	0.5 ± 0.2	0.206	0.5 ± 0.1	0.6 ± 0.5	0.096	0.5 ± 0.3	0.8 ± 1.2	0.305
β-Aminoisobutyric Acid	9.5 ± 11.2	9.9 ± 15.5	0.916	9.8 ± 11.9	8.5 ± 11.6	0.661	9.9 ± 12.7	11.6 ± 17.2	0.661
α-Aminobutyric acid	22.6 ± 11.1	16.9 ± 9.9	0.026	22.1 ± 8.8	18.1 ± 12.9	0.143	21.7 ± 9.9	22.5 ± 16.4	0.812
Homocitrulline	1.6 ± 2.0	2.6 ± 2.3	0.085	1.6 ± 1.7	2.9 ± 2.6	0.019	1.4 ± 1.4	3.4 ± 3.1	0.003
Homocystine	0.7 ± 0.3	0.7 ± 0.5	0.657	0.7 ± 0.3	0.6 ± 0.2	0.468	0.8 ± 0.5	1.0 ± 1.7	0.410
Kynurenine	17.8 ± 10.4	31.7 ± 22.6	0.002	19.4 ± 11 .1	39.5 ± 30.6	0.001	16.8 ± 11.0	42.2 ± 52.7	0.014

Alloisoleucine	2.0 ± 1.3	1.3 ± 0.5	0.008	1.9 ± 1.5	1.3 ± 0.6	0.053	1.4 ± 0.4	1.6 ± 1.2	0.458

Values are presented as mean ± standard deviation. ARDS: acute respiratory distress syndrome.
